# Targeted delivery of oral vaccine antigens to aminopeptidase N protects pigs against pathogenic *E. coli* challenge infection

**DOI:** 10.3389/fimmu.2023.1192715

**Published:** 2023-06-29

**Authors:** Hans Van der Weken, Hamid Reza Jahantigh, Eric Cox, Bert Devriendt

**Affiliations:** ^1^ Laboratory of Immunology, Department of Translational Physiology, Infectiology and Public Health, Faculty of Veterinary Medicine, Ghent University, Merelbeke, Belgium; ^2^ Department of Pathology, Faculty of Medicine, Emory University, Atlanta, GA, United States; ^3^ Interdisciplinary Department of Medicine – Section of Occupational Medicine, University of Bari, Bari, Italy

**Keywords:** oral vaccination, challenge infection, E. coli, aminopeptidase N, epithelial targeting, mucosal immunity, recombinant antibody, subunit vaccine

## Abstract

Oral subunit vaccines are an interesting alternative strategy to traditional live-attenuated or inactivated vaccines for conferring protection against gut pathogens. Despite being safer and more cost-effective, the development of oral subunit vaccines remains challenging due to barriers imposed by the gastrointestinal tract, such as digestive enzymes, a tolerogenic immune environment and the inability of larger proteins to cross the epithelial barrier. Recent advances have focused on overcoming these barriers by using potent mucosal adjuvants or pH-responsive delivery vehicles to protect antigens from degradation and promote their release in the intestinal lumen. A promising approach to allow vaccine antigens to pass the epithelial barrier is by their targeting towards aminopeptidase N (APN; CD13), an abundant membrane protein present on small intestinal enterocytes. APN is a peptidase involved in digestion, but also a receptor for several enteric pathogens. In addition, upon antibody-mediated crosslinking, APN facilitated the transport of antibody-antigen fusion constructs across the gut epithelium. This epithelial transport resulted in antigen-specific immune responses. Here, we present evidence that oral administration of APN-specific antibody-antigen fusion constructs comprising the porcine IgA Fc-domain and the FedF tipadhesin of F18-fimbriated *E. coli* elicited both mucosal and systemic immune responses and provided at least partial protection to piglets against a subsequent challenge infection with an F18-fimbriated STEC strain. Altogether, these findings will contribute to the further development of new oral subunit vaccines and provide a first proof-of-concept for the protective efficacy of APN-targeted vaccine antigens.

## 1 Introduction

Oral subunit vaccines hold great promise to protect against gut pathogens in a safer and more cost-effective manner than traditional live-attenuated or inactivated vaccines. However, the development of oral subunit vaccines remains challenging due to the barriers imposed by the gastrointestinal tract. The presence of digestive enzymes, the tolerogenic immune environment pervading the gut and the inability of larger proteins to cross the gut epithelial barrier all contribute to a poor bioavailability and immunogenicity of oral subunit vaccines. Several strategies have been developed in recent years to overcome these barriers, including the use of potent mucosal adjuvants to circumvent the tolerogenic environment and pH-responsive delivery vehicles, such as nanoparticles, that protect the vaccine antigens from degradation in the gastrointestinal tract and promote their release in the intestinal lumen (1, 2).

Despite these advances, a major issue holding back the development of new oral subunit vaccines is their inability to cross the small intestinal epithelial barrier. As a result, most oral vaccine candidates induce weak mucosal immune responses. One promising approach to overcome this challenge is to target the vaccine antigens towards receptors present on the apical side of gut epithelial cells that facilitate transport across the gut epithelium (3). Two examples include glycoprotein 2 (GP2) present on Peyer’s patch M cells and the neonatal Fc receptor (FcRn) present on absorptive enterocytes. GP2 specifically recognizes FimH, a component of type I pili of certain Gram-negative enterobacteria, and promotes the uptake of FimH^+^ bacteria, resulting in specific mucosal immune responses in mice (4). Oral administration of a biotinylated ovalbumin peptide conjugated with an anti-GP2-streptavidin fusion antibody was able to induce ovalbumin-specific mucosal immune responses in mice by targeting the peptide towards GP2 (5). One downside of targeting M-cells is their relative low abundance in the gut epithelium. In contrast, enterocytes are by far the most abundant epithelial cell type in the intestine and express FcRn. This receptor interacts with the Fc domain of IgG in a pH-specific manner and allows for bi-directional transport through the intestinal epithelium (6–9). Oral administration of IgG Fc domain-coupled prepro-insulin in mice resulted in efficient transport through the intestinal epithelium and was taken up by antigen-presenting cells and transported to the spleen and thymus (10). Furthermore, oral delivery of recombinant *Lactobacillus plantarum* expressing the influenza viral protein M2e fused to an IgG Fc domain resulted in protective immunity against subsequent infection with influenza viruses in mice (11).

Another attractive target receptor expressed by small intestinal epithelial cells is aminopeptidase N (APN; CD13). This highly glycosylated, homodimeric membrane protein plays a role in cholesterol uptake and in the final digestion of peptides (12, 13). APN is also expressed on conventional dendritic cells, where it plays a role in antigen processing and presentation (14). Due to its highly conserved nature across different species, including pig and human, it represents an interesting target for the oral delivery of vaccine antigens. We previously demonstrated that antibody-mediated targeting of antigens and microparticles to APN triggered their transcytosis through the gut epithelial barrier (15–18). This resulted in their uptake by small intestinal antigen-presenting cells, subsequent transport to the mesenteric lymph nodes and the induction of robust intestinal IgA responses. Recently, we fused the FedF tipadhesin from F18-fimbriated *E. coli* to porcinized APN-specific monoclonal antibodies. Upon oral administration to piglets both systemic and intestinal FedF-specific antibody responses were elicited. However, it remained unresolved whether these immune responses were sufficient to protect animals against infection with F18-fimbriated *E. coli* (15).

Post-weaning diarrhea and edema disease are important causes of illness in recently weaned piglets, leading to growth retardation, mortality and significant economic losses. The primary causative agents of these diseases are F4- and F18-fimbriated enterotoxigenic *Escherichia coli* (ETEC) and F18-fimbriated Shiga-toxin producing *E. coli* (STEC) strains (19). Current strategies for preventing infections in weaned piglets rely on good sanitation practices and the use of antimicrobial agents, such as antibiotics and zinc oxide. Due to concerns on increased antibiotic resistance, the preventive use of antibiotics in the pig industry has been banned in Europe since 2006, while the use of zinc oxide has also been restricted since 2022. For these reasons, the development of alternative strategies, like vaccines, to prevent disease is of utmost importance (20, 21). Currently, a live oral vaccine against F4- and F18-fimbriated ETEC, Coli-protec, is marketed (22). While live vaccines are efficient at preventing disease, some concerns have been raised on uncontrolled replication, severe inflammatory reactions, and the risk of reversion to virulence (23, 24). Additionally, the use of live vaccines precludes their use with other interventions that are aimed at preventing bacterial infections during the post-weaning period, such as antimicrobial compounds or feed supplements. For these reasons, there is currently a high need for the development of new oral vaccination strategies to prevent these bacterial infections in weaned piglets. Here, we further investigated the protective efficacy of our APN targeted vaccine candidate by challenging immunized piglets with an F18-fimbriated STEC strain.

## 2 Methods

### 2.1 Production of recombinant antibodies

The chimeric αAPN-pIgA-FedF fusion antibody was generated as previously described, using the variable regions of the porcine APN-specific IMM013 clone (mouse antibody) and the porcine constant light (AAA03520.1) and porcine IgA heavy (AAA65943.1) chains. The heavy chain was genetically fused to the tipadhesin FedF_15-165_ of F18 fimbriae (PDB entry: 4B4P) using a (G_4_S)_3_-flexible linker (25). Recombinant antibodies were secreted by CHO and subsequently purified using ammonium sulphate precipitation between 43 and 47% saturation and dialyzed against PBS. The final formulation of the purified product contained 600 µg/ml of the αAPN-pIgA-FedF fusion antibody and 20 mg/ml BSA, which serves as a decoy protein for proteolytic degradation in the small intestine (**Figure S1**).

### 2.2 Animals and immunization procedures

Sixteen conventionally reared piglets (Belgian Landrace x Pietrain) from a Belgian farm were weaned at 3 weeks and transported to our facilities. These animals were screened to be F18 fimbriae seronegative and F18 receptor positive using FUT1 genotyping (26). The piglets were housed in isolation units and treated with colistin (Colivet quick pump^®^, 6.4 mg/kg bodyweight) for 5 days before the start of the experiment. Animals were randomly divided in 2 groups of 8 animals and housed in a single unit. The piglets were orally immunized for 3 consecutive days, followed by a booster immunization 14 days post primary immunization (dppi). The gastric pH was neutralized by administration of Omeprazole (20 mg) 24 hours before each immunization and animals were deprived of feed and water 3 hours before and 1 hour after each immunization. Animals were immunized by oral administration with a syringe containing 3 mg of the recombinant αAPN-pIgA-FedF fusion antibody, 10 mg BSA as a decoy protein and adjuvanted with 50 µg cholera toxin (Merck, C8052) in 10 ml PBS for the vaccine group or 10 ml PBS for the control group. Blood was collected at 0, 14, 21, 28, 35, 42 and 49 dppi to analyze antigen-specific serum responses. The animals were euthanized at 49 dppi by intravenous injection of sodium pentobarbital 20% (60 mg/2.5 kg BW; Kela) and upon exsanguination, intestinal content was collected from the ileum for detection of antigen-specific IgA antibodies.

### 2.3 Challenge infection

Piglets were infected with an F18-fimbriated Shiga toxin-producing *Escherichia coli* (STEC) strain 2 weeks after the booster immunization (28 dppi). Therefore, the piglets were first sedated intramuscularly with Stressnil (2 mg/kg body weight), after which the pH of the stomach was neutralized with 62 ml NaHCO_3_ (1.4% w/v; intragastric administration). A half hour later, piglets were infected with 10^11^ F18^+^ STEC (F107/86 strain (O139:H1; F18ab^+^; Stx2e^+^; Streptomycin-resistant) in 10 ml PBS. The piglets were deprived of feed and water 3 hours before and 2 hours after the infection. Feces were subsequently collected for 12 consecutive days to monitor bacterial excretion. Therefore, fecal serial dilutions (5 to 0.00001%; w/v) were made in sterile PBS and plated onto blood agar (BBL™ Blood agar base infusion agar; BD Biosciences) plates containing 1 mg/ml streptomycin.

F18 fimbriae expression by the colonies was confirmed by dot blot. Briefly, PVDF membranes (Amersham™ Hybond™; Cytiva) were incubated in methanol for 10 minutes, washed in UP water, placed on the colony-containing bacterial plates and incubated for 2 hours. After an overnight blocking step in PBS + 5% milk + 0.2% Tween-80, the membranes were subsequently incubated for 1 hour at room temperature with a FedA-specific mouse monoclonal antibody (IMM02; in-house), followed by a 1 h incubation step with an anti-mouse IgG-HRP (P0260, dako). Membranes were washed with PBS 3 times for 5 min in between each incubation step. Positive colonies were subsequently detected by developing the membrane with 3-amino-9-ethylcarbazole (AEC). The reaction was stopped with UP water.

### 2.4 FedF-specific immune responses

Blood was collected from the jugular vein into a gel and clot activator tube (Vacutest, Kima). After 1h incubation at RT, tubes were centrifuged and serum was collected, inactivated at 56°C for 30 minutes and treated with kaolin to reduce background levels in ELISA. Serum samples were stored at -20°C until use. After euthanasia and exsanguination, intestinal content was collected from the ileum (0.25 g) and further homogenized in 5 ml ice-cold extraction buffer (0.1% BSA, 0.05% Tween-20 and Complete protease inhibitor cocktail (Sigma)) using glass beads. The supernatant (4 ml) was subsequently mixed with 1.25 ml glycerol and heated for 10 minutes at 56°C after which the samples were snap-frozen in liquid N_2_ and stored at -20°C for further analysis.

Maxisorp microtiter plates (96-well, Life Technologies) were coated with FedF (5 µg/ml; in-house) in PBS for 2h at 37°C (18). After overnight blocking at 4°C with PBS + 3% BSA + 0.2% Tween-80, different dilutions of serum or intestinal content were added in dilution buffer (PBS + 3% BSA + 0.2% Tween-20) to the wells. The serum was serially diluted starting at 1/25 dilution, while the intestinal content was diluted 1/2. After incubation for 1 h at 37°C, plates were washed and incubated for 1 h with HRP-conjugated mouse anti-pig IgG (1/1000; MabTech) or IgA (1/10000; Bethyl). Following 3 washes, ABTS was added and the optical density was measured at 405nm after 60 minutes incubation at 37°C using a spectrophotometer (Tecan SpectraFluor).

### 2.5 *In vitro* villous adhesion assay

An *in vitro* adhesion assay on small intestinal villi was performed as described previously (27). Briefly, jejunal villi were collected at euthanasia for all piglets and the binding of the F18-fimbriated STEC strain F107/86 to the villi was tested by adding 4x10^8^ bacteria to an average of 50 villi in 500 µl PBS while gently shaking for 1h at room temperature. The villi were subsequently examined by phase-contrast microscopy at a 600x magnification. The mean number of bacteria adhering to the brush border were counted for 15 randomly selected places of 50 µm in length for each piglet (**Figure S2**).

### 2.6 Ethical statement

All animal procedures were reviewed and approved by the Ethical Committee of the Faculty of Veterinary Medicine of Ghent University (EC2021-025).

### 2.7 Data analysis

The data were analyzed using Graphpad Prism software version 9. Serum IgG responses and bacterial excretion numbers were analyzed using two-way ANOVA (mixed-effects model) with repeated measures. IgA responses between groups in the ileal content were analyzed using the Mann-Whitney test. Homogeneity of variances was assessed with Levene’s test. Multiple comparisons were corrected using the Two-stage linear step-up procedure of Benjamini, Krieger and Yekutieli. Differences were considered significant when the adjusted p-value <.05.

## 3 Results

### 3.1 Systemic and local immune responses after oral immunization with APN-targeted antigen

**Figure 1:**
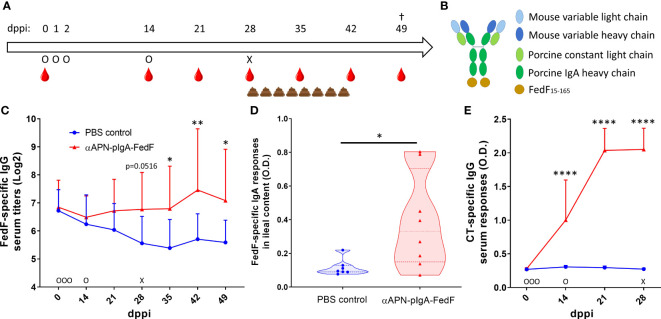
Increased FedF-specific immune responses after oral immunization with an APN-specific antibody-antigen fusion construct. **(A)** Timeline of the experiment with days of oral immunization (O), challenge infection with an F18-fimbriated STEC strain (X), feces collection and blood collection time points. **(B)** schematic structure of the antibody-antigen fusion construct. **(C)** FedF-specific IgG serum responses from 0 to 49 days post primary immunization (dppi) and **(D)** FedF-specific IgA antibodies in the ileal content at the day of euthanization (49 dppi). **(E)** CT-specific IgG serum responses from 0 to 28 dppi. *Indicates a significant difference compared to the PBS control group. *: p <.05, **: p <.01, ****: p <.0001, n = 8 per group.

To evaluate the ability of the APN-targeted antibody-antigen fusion construct to provide protection against infection, a challenge infection experiment was performed (**Figure 1A**). To this end, piglets (n=8 per group) received orally the chimeric APN-specific porcine IgA-FedF fusion construct (**Figure 1B**; αAPN-pIgA-FedF) adjuvanted with cholera toxin in a prime-boost regime. Animals in the control group received PBS. The ability to elicit FedF-specific systemic and local immune responses was subsequently evaluated by ELISA (**Figure 1C, D**). Here, we showed increased FedF-specific serum IgG responses 28, 35, 42 and 49 days post primary immunization (dppi) for the APN-targeted fusion construct as compared to the control group (**Figure 1C**). No increased FedF-specific serum IgA responses were observed (data not shown). More importantly, FedF-specific IgA antibodies were increased in the ileal content (49 dppi) of piglets orally immunized with the APN-targeted fusion construct in comparison with the control group (**Figure 1D**). To confirm effective delivery of the oral vaccine, the IgG serum response against the adjuvant cholera toxin was also evaluated. As shown in **Figure 1E**, a strong increase could be observed for the immunized animals compared to the control group starting from 14 dppi (**Figure 1E**). Since the vaccine construct contained the Fc domain of pig IgA and CT is a potent mucosal adjuvant, we wondered whether antibody responses against pig IgA were induced. Using ELISA, pig IgA-specific IgG serum responses were not observed (**Figure S3**). These data show that the oral immunization with the APN-targeted FedF primed the immune system, which boosted the FedF-specific systemic and local immune responses upon challenge infection with an F18-fimbriated *E. coli* strain.

### 3.2 Rapid reduction and clearance of F18+ STEC after challenge infection

**Figure 2:**
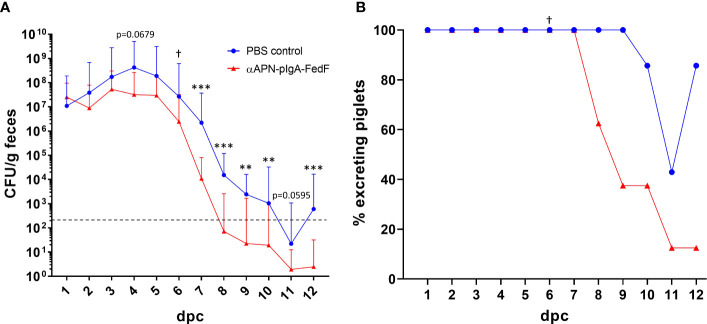
Rapid reduction and clearance of the pathogen after challenge infection. **(A)** Mean bacterial excretion over time after a challenge infection with an F18-fimbriated STEC strain. Error bars represent the standard deviation. The dashed line represents the detection limit (200 CFU/g feces). **(B)** The percentage of piglets with detectable excretion levels of the F18+ STEC strain over time. CFU: Colony forming units. dpc: days post challenge. *Indicates significant differences compared to the control group. **: p <.01, ***: p <.001. †one piglet in the control group was euthanized due to severe symptoms of edema disease. n = 8 per group.

To assess whether the induced immune responses were sufficient to provide protection against infection, the piglets were challenged with an F18-fimbriated STEC strain at 28 dppi (**Figure 1A**). The bacterial excretion was monitored for 12 consecutive days. Here, a significant reduction (200-fold) in the bacterial excretion between the immunized animals and the control group could be observed starting from 7 days post challenge (dpc) (**Figure 2A**). Furthermore, the excretion levels of the infection strain dropped below the detection limit (2x10^2^ CFU/g feces) in 62.5% of piglets in the immunization group at 9 dpc, compared to none of the piglets in the control group (**Figure 2B**). At the end of the fecal collection period (12 dpc), the percentage of piglets where the infection strain was no longer detectable increased to 87.5% for the immunized animals as opposed to 14.3% for the control group. These data clearly show that the immune responses elicited by oral administration of the APN-targeted antibody-antigen vaccine construct resulted in a more rapid reduction and clearance of the pathogen and partially protected the piglets from infection. This is further highlighted by the observation of severe symptoms of edema disease in one piglet of the control group, which had to be euthanized at 6 dpc. All animals were shown to be susceptible to F18^+^ *E. coli* infection postmortem using a villous adhesion assay.

## 4 Discussion

We have previously identified APN as an interesting target for the oral delivery of vaccine antigens, as binding of APN-specific antibodies towards APN leads to transcytosis through the intestinal epithelium and subsequent uptake by antigen-presenting cells. Targeting of the tipadhesin FedF, a protective antigen for F18-fimbriated *E. coli*, to APN using chimeric porcinized IgA antibody-antigen fusion constructs led to both systemic and mucosal immune responses after oral delivery and gave a first indication that APN targeting could be used for the development of new oral subunit vaccines (15–18).

In this study, we further explored the potential of APN targeting in oral vaccination and showed that the observed immune responses were sufficient to confer at least partial protection against infection by an F18-fimbriated STEC strain. A 200-fold reduction in bacterial excretion could be observed 7 days after the challenge infection for immunized piglets in comparison with control animals. Furthermore, the immunized piglets were able to clear the pathogen more rapidly. In 62.5% of the immunized piglets, the infection pathogen could no longer be detected 9 days after infection, increasing to 87.5% at the end of the fecal collection period (day 12). On the other hand, in the control group, not a single animal was able to clear the pathogen on day 9 and only one animal had completely cleared the pathogen at the end of the experiment. These results clearly indicate that the immunized animals were partially protected against infection. Furthermore, only in the control group, one animal suffered from severe symptoms of edema disease and had to be euthanized. Although morbidity rates can vary a lot between strains and usually remain low, mortality rates of affected piglets can go as high as 50 to 90% (28–31). Unfortunately, no firm conclusions can be drawn regarding protection against morbidity or mortality as the group sizes were too small and this was not the aim of the experiment. Further efforts should include a field trial to assess the efficacy of this vaccine candidate in preventing disease symptoms and mortality.

Although the induced immune responses were not sufficient to completely prevent pathogen colonization, this is not necessary to protect infected animals from severe disease symptoms and mortality. As was shown by Nadeau et al., a strong reduction in bacterial excretion levels can be sufficient to reduce the infection burden and resulting symptoms of ETEC/STEC infection (22). In that study, animals were orally vaccinated with the live-attenuated Coli-protec vaccine and infected with an F18-fimbriated *E. coli* strain, producing multiple toxins (9910297-^2STM^; STb^+^, LT^+^, East-1^+^, Stx2e^+^, F18ab^+^). Their results indicated that a reduction to around 10^6^ CFU/g stool seemed to be sufficient to prevent most signs of infection, including mild to moderate diarrhea. In our experiment, the infection burden peaked within the first 5 days of infection, after which excretion levels dropped drastically. This indicates the presence of local immune responses sufficient to clear the pathogen. Although not significant, the peak excretion levels for the immunized animals also appear to be lower compared to the control animals, which reached peak excretion levels exceeding 10^8^ CFU/g feces for three consecutive days. A further reduction of the peak bacterial excretion in the vaccinated animals by a factor 10 would result in excretion levels reaching 10^6^ CFU/g, which would be similar to the results of Nadeau et al. and should be sufficient to prevent disease symptoms. Of note, vaccines that fail to completely block transmission of the target pathogen might be a risk for the emergence of vaccine-escape mutants.

The observed reduction in bacterial excretion could be further strengthened by increasing the local antibody immune response against the vaccine antigen. It is known that FedF has a poor immunogenicity and we previously showed this can be attributed to its immune suppressive properties by decreasing the antigen-presenting capacity of intestinal antigen-presenting cells (15). This is further highlighted by the fact that no significant increases in FedF-specific serum IgG and IgA in the ileal content could be observed in the control group 3 weeks after the challenge infection. Besides targeting towards APN, the immunogenicity of FedF could be further improved by multimerization. This strategy is known to promote antigen recognition of low affinity B-cells and promote their differentiation to antibody-secreting plasma cells (32). What is also worth considering is that subunit vaccines based on a single antigen might fail to provide sufficient protection, even if strong immune responses to the target antigen are induced. Therefore, it might be interesting to develop a multivalent vaccine. In particular for targeting F18-fimbriated ETEC/STEC infections, the major fimbrial subunit FedA might provide another interesting candidate as a vaccine antigen. A multivalent vaccine approach would also have the advantage that different strains can be targeted. For example, by combining the FaeG subunit of F4 fimbriae and the FedF subunit of F18 fimbriae, a multivalent subunit vaccine targeting the two most common causative agents of post-weaning diarrhea and edema disease could be developed. Our vaccine candidate contained cholera toxin (CT). While CT is a potent mucosal adjuvant, it is also toxic to humans. As such, adjuvating our vaccine candidate with CT might pose a risk for personnel and detoxified mmCT might be a better alternative (33).

Because our vaccine candidate uses a porcine IgA Fc domain, the antibody-antigen fusion construct might be recognized by FcαRI (CD89) expressed by myeloid cells, such as monocytes, macrophages and specific dendritic cell subsets (34, 35). FcαRI binds IgA at the CH2-CH3 interface. While the FedF_15-165_ antigen is fused to the CH3 domain, it most likely does not block recognition of IgA by FcαRI, as fusing FedF to mouse IgG1 did not interfere with protein G-mediated purification (15). Protein G binds the Fc domain of IgG at the CH2-CH3 interface. Since binding of monomeric IgA to FcαRI can elicit an inhibitory signaling cascade in FcαRI-expressing immune cells, this interaction could result in a reduced immunogenicity of our vaccine candidate (36). However, FcαRI is not yet well characterized in pigs. Further research is thus needed to elucidate whether our constructs elicit FcαRI-mediated signaling in porcine immune cells and whether this synergizes with APN-mediated signaling.

In conclusion, the results presented here provide evidence for APN targeting as a promising new technology for delivery of vaccine antigens to the gut immune system and will contribute to the development of new and effective oral subunit vaccines. If the immunogenicity of FedF can be further improved, this could lead to the development of a novel subunit vaccine capable of protecting piglets against edema disease. Furthermore, as APN is a highly conserved protein, this vaccine delivery technology could be translated to other species as well, including humans.

## Data availability statement

The raw data supporting the conclusions of this article will be made available by the authors, without undue reservation.

## Ethics statement

The animal study was reviewed and approved by Ethical Committee of the Faculty of Veterinary Medicine of Ghent University.

## Author contributions

BD and EC conceived the idea and designed the research. HV and HJ produced and purified the recombinant antibodies. HV performed the *in vivo* experiment. HV performed the data analysis together with BD and EC. HV wrote the manuscript with contributions from BD and EC. All authors reviewed the manuscript before submission. All authors contributed to the article and approved the submitted version.

## Supplementary material

Supplementary Figure 1 Purity of the porcine IgA-FedF fusion constructs. Coomassie staining (left) and BSA-specific western blot (right) of the vaccine candidate after ammonium sulphate precipitation and dialysis in PBS. DC: Precision Plus Protein Dual Color Standard (Bio-rad). MM: MagicMark™ XP Western Protein Standard (Thermo Fisher).

Supplementary Figure 2
*In vitro* villous adhesion assay. The average number of bacteria adhering to the brush border of the jejunal villi was calculated for each piglet after incubation with an F18-fimbriated STEC strain (F107/86). The dotted line indicates the susceptibility threshold (5 bacteria/250 µm villi). Numbers on the x-axis indicate the individual piglets.

Supplementary Figure 3 No pig IgA-specific IgG antibodies could be observed in the pig serum after immunization. A porcine IgA-specific ELISA was performed by coating a Maxisorp plate with a polyclonal anti-pig IgA antibody (10 µg/ml), followed by IgG-depleted pig serum (diluted to 10 µg/ml porcine IgA). The binding of the porcine IgA from the serum to the capture antibody was confirmed using an HRP-conjugated anti-pig IgA antibody. After blocking with BSA (1%), serum (1/10 dilution) from the different piglets (n = 8 per group) at 0 and 28 days post primary immunization (dppi) was incubated and detected with an HRP-conjugated anti-pig IgG antibody. The optical density (O.D.) was measured at 405nm after 60 min incubation with ABTS at 37°C. In between each incubation, three wash steps were performed with PBS + 0.2% Tween-20. Data is shown as the average ± standard deviation.
