# Supplementary figures and images for: Targeted delivery of oral vaccine antigens to aminopeptidase N protects pigs against pathogenic E. coli challenge infection
Source: Front Immunol. 2023 Jun 29;14:1192715. doi: 10.3389/fimmu.2023.1192715 (PMC10338862; doi:10.3389/fimmu.2023.1192715)

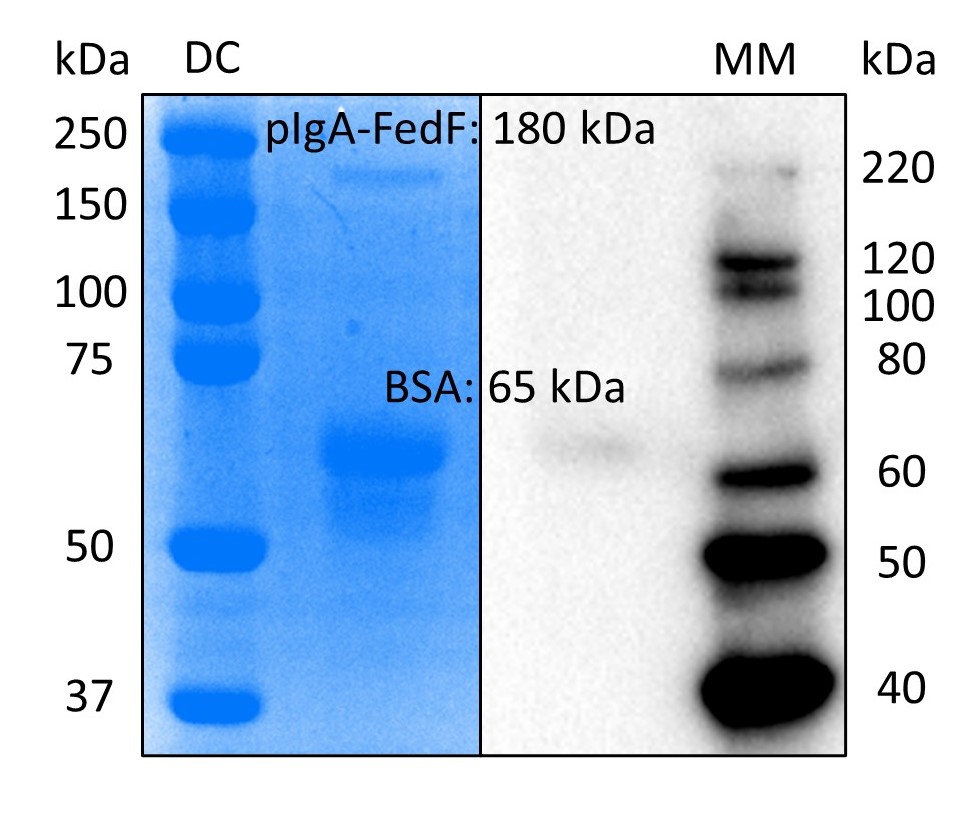

Supplement: Supplementary Figure 1 — Purity of the porcine IgA-FedF fusion constructs. Coomassie staining (left) and BSA-specific western blot (right) of the vaccine candidate after ammonium sulphate precipitation and dialysis in PBS. DC: Precision Plus Protein Dual Color Standard (Bio-rad). MM: MagicMark™ XP Western Protein Standard (Thermo Fisher). [file Image_1.jpeg]

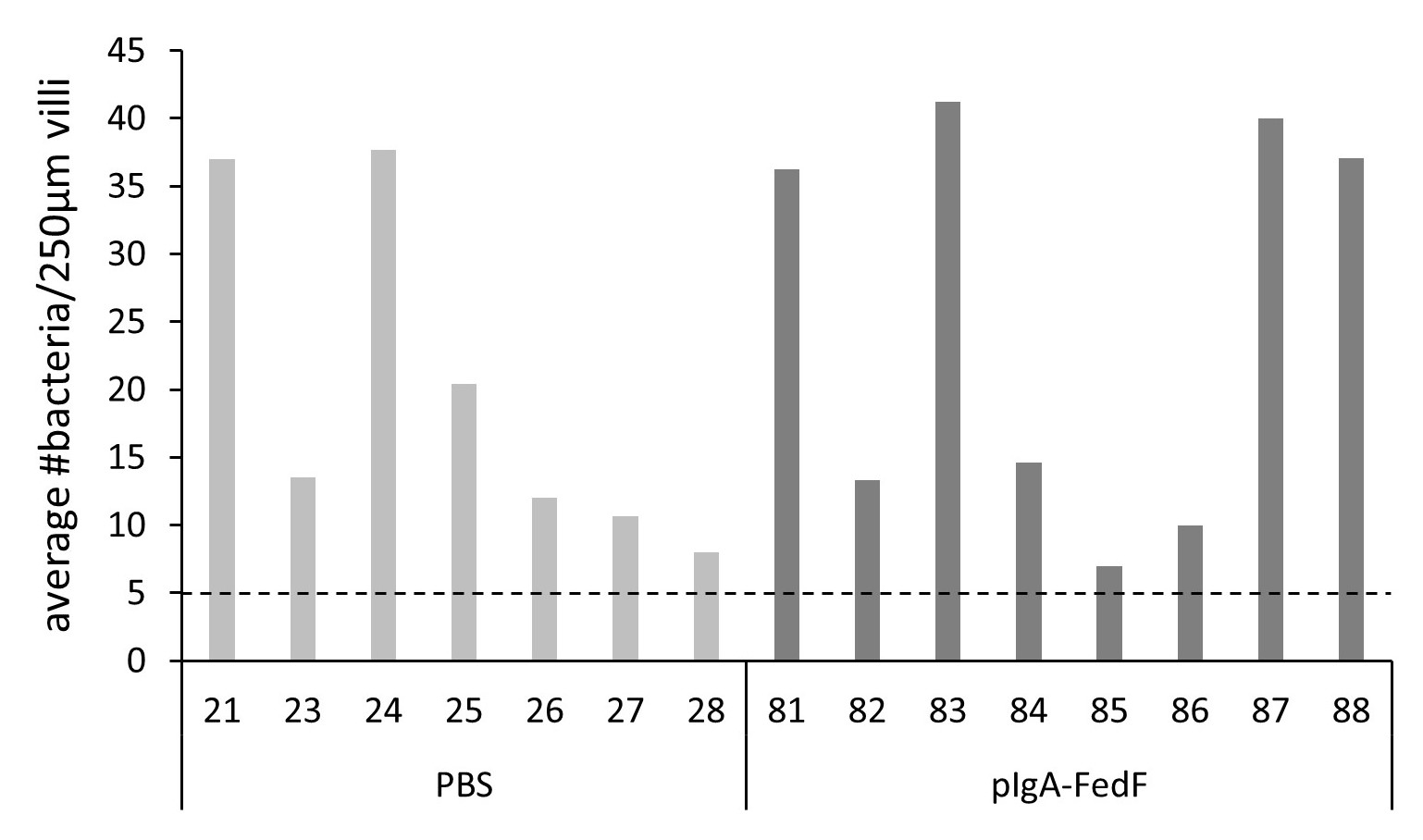

Supplement: Supplementary Figure 2 — In vitro villous adhesion assay. The average number of bacteria adhering to the brush border of the jejunal villi was calculated for each piglet after incubation with an F18-fimbriated STEC strain (F107/86). The dotted line indicates the susceptibility threshold (5 bacteria/250 µm villi). Numbers on the x-axis indicate the individual piglets. [file Image_2.jpeg]

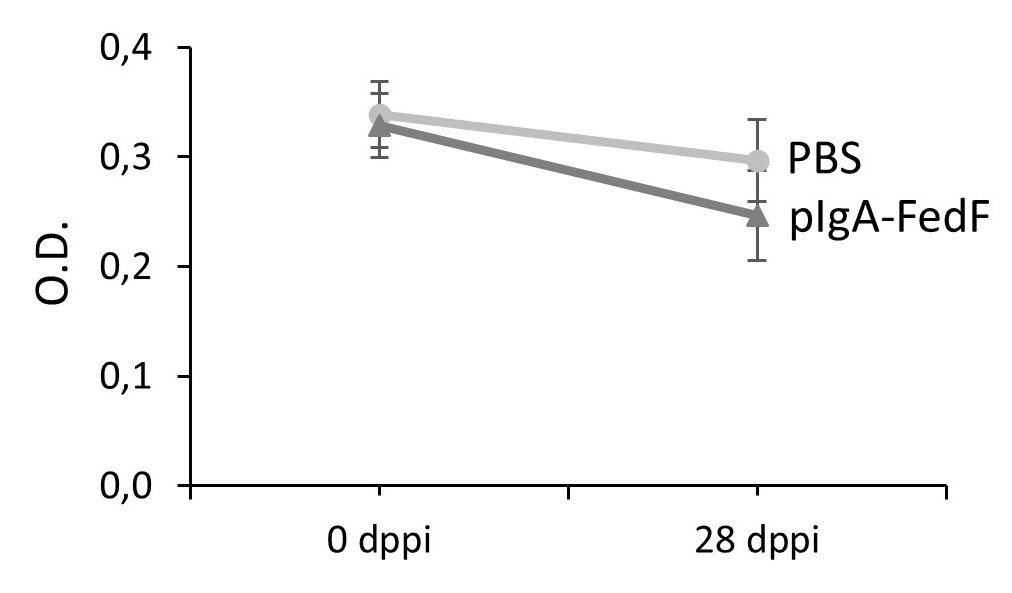

Supplement: Supplementary Figure 3 — No pig IgA-specific IgG antibodies could be observed in the pig serum after immunization. A porcine IgA-specific ELISA was performed by coating a Maxisorp plate with a polyclonal anti-pig IgA antibody (10 µg/ml), followed by IgG-depleted pig serum (diluted to 10 µg/ml porcine IgA). The binding of the porcine IgA from the serum to the capture antibody was confirmed using an HRP-conjugated anti-pig IgA antibody. After blocking with BSA (1%), serum (1/10 dilution) from the different piglets (n = 8 per group) at 0 and 28 days post primary immunization (dppi) was incubated and detected with an HRP-conjugated anti-pig IgG antibody. The optical density (O.D.) was measured at 405nm after 60 min incubation with ABTS at 37°C. In between each incubation, three wash steps were performed with PBS + 0.2% Tween-20. Data is shown as the average ± standard deviation. [file Image_3.jpeg]
